# Experience Summary of Laparoscopic Treatment for Pediatric Ureteral Polyps

**DOI:** 10.3389/fped.2021.689842

**Published:** 2021-06-30

**Authors:** Hongjie Gao, Jiawei Chen, Guowei Li, Xinhai Cui, Fengyin Sun

**Affiliations:** ^1^Department of Pediatrics, Qilu Hospital of Shandong University, Jinan, China; ^2^Department of Pediatric Surgery, Qilu Hospital of Shandong University, Jinan, China

**Keywords:** laparoscopy, ureteral polyps, multiple polyps, children, experience summary

## Abstract

**Objective:** To investigate surgical techniques and challenges of laparoscopic in treating pediatric ureteral polyps under laparoscopy.

**Methods:** The clinical data of 7 of pediatric ureteral polyps patients who were admitted to the hospital from July 2015 to January 2020 were analyzed retrospectively. There were 6 males and 1 female from 7.7 to 13.9 years old at the mean age of 10.4. Before surgery, all children performed urinary B ultrasound, magnetic resonance urography (MRU), and renal radionuclide scanning. Six cases were observed on the left lateral and 1 on the right. The lesions of 5 cases were located at the ureteropelvic junction, 1 in the upper ureter and 1 in the middle ureter. The polyps were treated intraoperatively by the resecting of the lesion segment and simple polypectomy to retain the attached part of the original diseased segment of the ureter. All surgeries were performed under laparoscopy and B-ultrasound was performed during follow up after surgery.

**Results:** All 7 surgeries were performed successfully under the laparoscope. The surgery time was 80–110 min, and the average surgery time was 97.5 min. The intraoperative bleeding was 10–25 ml and the average postoperative hospital stay was 6 d. Postoperative hematuria occurred in 1 case. Neither urinary leakage nor urinary tract infection was reported post surgery. Preoperative affected pyelectasis of all patients was 2.0–3.7 cm. Three months postoperatively, the affected pyelectasis was measured at 1.2–3.0 cm. No recurrence of polyps was reported after surgery. During the follow-up to April 2020, there was no significant change in the kidney size of all patients, and hydronephrosis was alleviated compared with that before surgery.

**Conclusions:** Laparoscopy is a safe, effective and minimally invasive surgical technique for pediatric multiple ureteral polyps. The surgery plan was designed according to the location and size of polyps, including segmental ureterectomy of polyps + pyeloureterostomy, segmental ureterectomy of polyps + ureter - ureteral anastomosis.

## Introduction

A ureteral polyp is a rare benign non-epithelial tumor derived from mesoderm, which can cause upper urinary tract obstruction (1). The traditional treatment recommends open surgical exploration and resection of polyps. As endoscopic technology and equipment advance, it has been reported that ureteral polyps have been successfully treated by ureteroscopy (2, 3). However, due to the narrow field of vision and small operating space under an endoscope, the surgery is especially difficult for long segmental polyps or huge polyps in children, which leads to incomplete resection of polyps or ureteral perforation or even avulsion. From July 2015 to January 2020, 7 cases of ureteral polyps were treated in this hospital under laparoscopy with satisfactory efficacy. The surgical technique and challenges are to be illustrated in the present paper.

## Clinical Data and Methods

### Clinical Data

**Figure 1:**
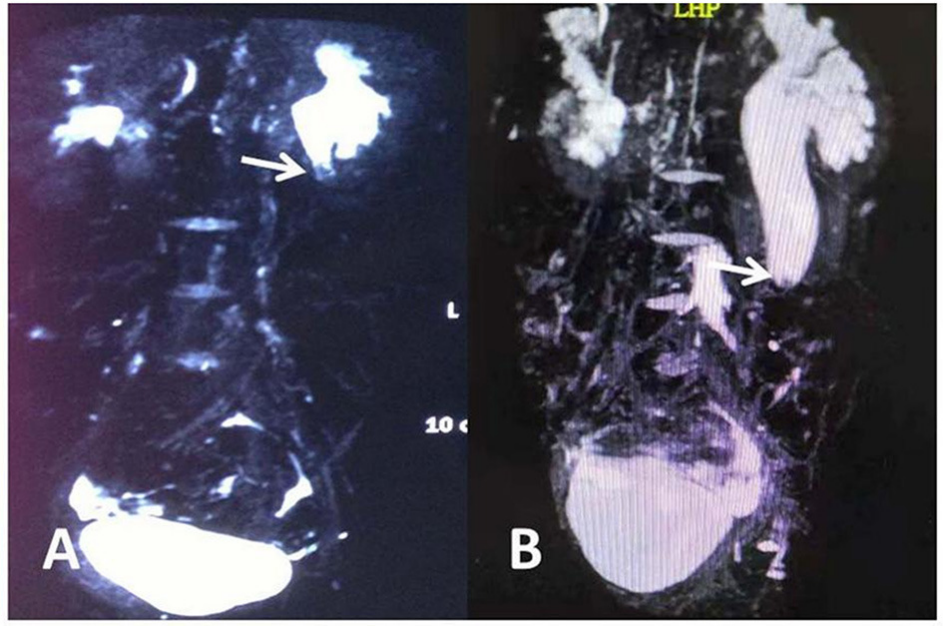
**(A)** Abnormal soft tissue can be seen at the ureteropelvic junction in MRU. **(B)** Nodular soft tissue is seen in the middle of the left ureter. The arrow indicates soft tissue shadow.

The clinical data of 7 cases of obstructive hydronephrosis caused by ureteral polyps from July 2015 to January 2020 were analyzed retrospectively. Before surgery, all children were examined by urinary B ultrasound, magnetic resonance urography (MRU) and renal radionuclide scanning which indicated the presence of varying degrees of hydronephrosis. Among them there were 6 males and 1 female from 7.7 to 13.9 years old at a mean age of 10.4. The lesions were located on the left in 6 cases and 1 case on the right. Five cases complained of low back pain and 2 with aggravated hydronephrosis were reported by pregnancy examination. Gross hematuria was found in 1 case and 1 case had urinary tract irritation. The case history ranged from 7 days to 5 years, with an average time of 9.4 months. Physical examination: All children had no abdominal masses, 5 cases had abdominal tenderness accompanied by percussion pain in the kidney area, and the rest had no obvious positive signs. The white blood cells in the urine of 2 children were positive. B ultrasound findings indicated severe hydronephrosis in 4 cases and moderate in 3 cases (Table 1). Preoperative magnetic resonance urinary (MRU) fluid imaging revealed that hydronephrosis was caused by either ureteral pelvic junction (UPJ) of the affected side or upper ureteral mass obstruction, and ureteral polyps were considered only two cases were reported accounting for 28% (2/7) (Figure 1). All children underwent renal radionuclide scanning to show the obstruction curve. The different renal function was 20–40%.

**Table 1:** Perioperative patient data.

	**Age (yrs)**	**Gender**	**Side**	**Clinlcal Symptom**	**MRU**
Patient 1	13.9	Male	left	Intermittent low back pain 5 years	The renal pelvis and calyces are significantly dilated and Ureteropelvic junction stenosis
Patient 2	11.5	Male	left	Ultrasound found left hydronephrosis 1 month	Abnormal soft tissue signal could be seen at the junction of renal pelvis and ureter
Patient 3	8.5	Male	right	Recurrent right abdominal pain 1 year	The right renal pelvis is significantly widened.
Patient 4	7.7	Male	left	Ultrasound found left hydronephrosis 1 week	Nodular soft tissue is seen in the middle of the left ureter
Patient 5	9.11	Male	lef	Upper Abdominal Pain 9months	Left caliceal are obviously dilated
Patient 6	8.3	Male	left	Left low back pain 1 week	Left hydronephrosis with UPJO
Patient 7	10.4	Femal	left	Abdominal Pain 1 month	Left hydronephrosis with ureteropelvic junction obstruction

*UPJO, ureteropelvic junction obstruction*.

### Surgical Methods

After anesthesia, the patients were placed in a lateral supine position at 45° on the healthy side and cushioned the waist on the affected side. A 5 mm Trocar was inserted directly through the umbilical natural cavity to establish pneumoperitoneum. Following laparoscopic insertion, the second and third 5 mm Trocars were inserted at the midpoint of umbilical and xiphoid, and the interior junction of ventral midline and lower ventral dermatoglyphics, respectively. Electrocoagulation hooks were utilized to open the posterior peritoneum and the fascia on the surface of the renal pelvis in a transmesocolonic approach, and the dilated renal pelvis or dilated ureter was subsequently exposed combining both blunt and sharp separation to locate the lesioned ureteral segment. A longitudinal incision was performed in the diseased segment of the lateral ureter, to explore and measure the length of the ureter invaded by polyps, and cut the ureter upward and downward until the normal segment free from polyps. The enlarged renal pelvis or normal ureter was full released according to the length of the lesioned ureter. On the premise of ensuring that there was no difficulty in the anastomosis, the polyps and their attached diseased segments of the ureter were removed completely. The lateral margin of the normal ureter was incised vertically ~1–1.5 cm to ensure the anastomosis of the enlarged renal pelvis or dilated ureter. A 5-0 absorbable suture was utilized for end-to-side intermittently anastomoses the trimmed lower renal pelvis pole or the proximal and distal ureteral. A 4.8 F double J stent was inserted into the ureter through the anastomosis, and the rest of the renal pelvis was sutured continuously.

### Surgical Findings

**Figure 2:**
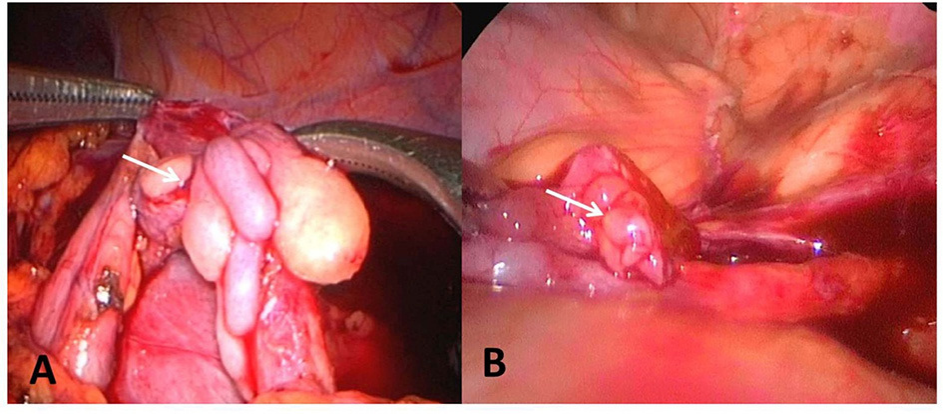
**(A)** The polyp is located at the ureteropelvic junction. **(B)** The polyp is located in the middle of the ureter and across the iliac artery. The arrow points to the polyp.

**Figure 3:**
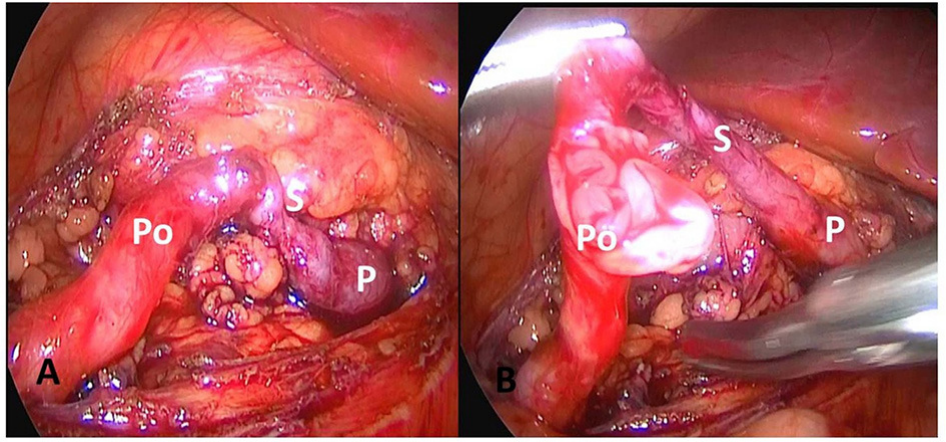
The polyp is located 2–3 cm from the upper to the renal pelvis, with proximal ureteropelvic junction stenosis. **(A)** Expose polyp and narrow ureter. **(B)** Open polyp segment ureter. P, pyelope; Po, polyp ureter; S, narrow ureter.

Five cases were reported that the ureteral wall at the ureteropelvic junction was markedly thickened, the polyp segmental ureter was thickened and tortuous, and moving mass was detected using a bending forceps, and the lesion was located at the ureteropelvic junction (Figure 2). Polyp segment ureterectomy and ureteropelvic anastomosis were performed. The polyp of one case of polyp was located at the upper end of the ureter at a distance of 2–3 cm away from the renal pelvis, complicated with stenosis of the proximal ureteropelvic junction (Figure 3). The polyp volume was about 1.3 ^*^ 1.2 ^*^ 0.7 cm, and the length of the segmental resection was about 2.5 cm. Excision of the polyp was performed including the regions of ureter close to the polyp. Polyp and stenosis ureterectomy and end-to-end anastomosis of renal pelvis and ureter were performed. The polyp reported in one case was located in the middle segmental ureter crossing the blood vessels and polyp segmental ureterectomy and uretero-ureteral end-to-end anastomosis was performed (Figure 2). The polypthe volume was about 1.5 ^*^ 1.5 ^*^ 0.5 cm with grayish white and the length of the resected lesion was about 1.8 cm.

**Figure 4:**
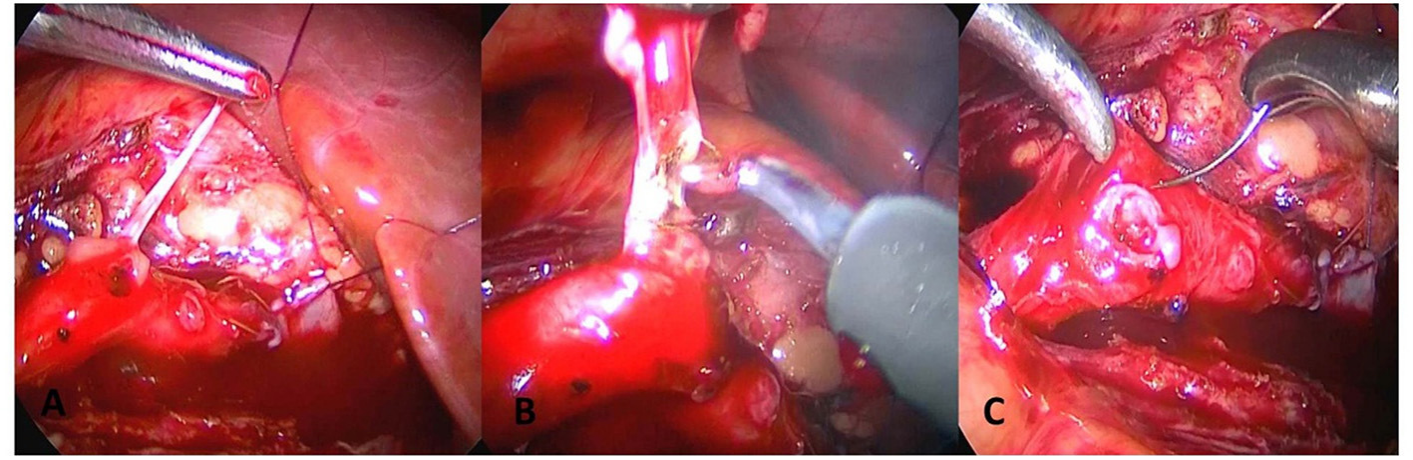
The polyp is only treated with complete polypectomy, and the ureter is perserved. **(A)** Longitudinal incision of ureter is performed to find long strip polyps. **(B)** Electric coagulation is performed to find long strip polyps. Electric coagulation is performed to remove polyps. **(C)** Transverse suture of ureter is performed.

Another case developed two starting points at the base of the polyp at a distance of 2 cm apart, and multiple polyps were considered. The first site was at the ureteropelvic junction and polypectomy and pyeloplasty were performed. The second polyp was subsequently observed. As reexcision of the lesion may be a challenge for anastomosis, the second polyp was only removed by simple polypectomy (Figure 4). The polyp volume was about 2.2 ^*^ 1.3 ^*^ 0.5 cm with vegetable shaped and the length of the resected lesion was about 2.8 cm.

**Figure 5:**
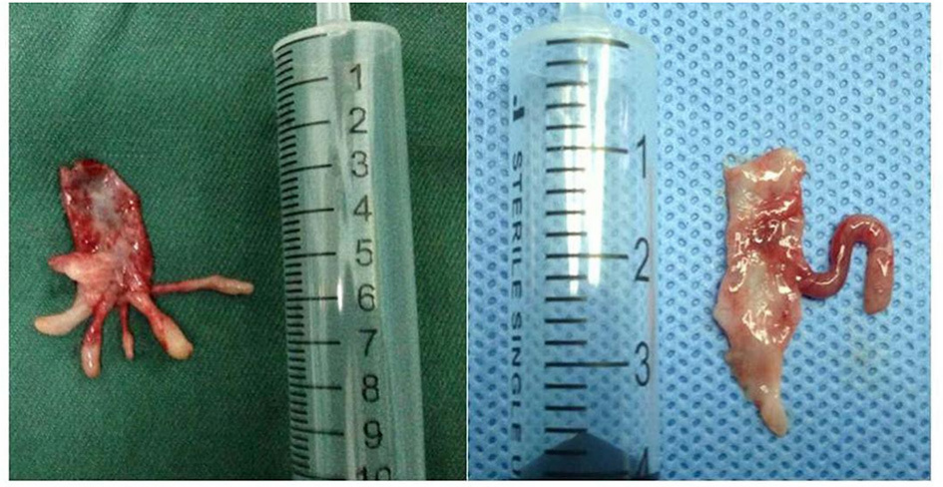
Polys look like anemone, with 1–5 branches, the longest of which was 2 cm.

Most of the polyps presented as anemone-like or octopus head-shaped, usually with 1–3 branches (Figure 5). The longest branch was 2 cm, and the shortest 2 mm. The branches of the polyps could float upward into the renal pelvis or downward extending to the ureter.

## Results

All 7 procedures were performed successfully under the laparoscope. The surgery time was 80–110 min, with an average of 97.5 min. The intraoperative bleeding was 10–20 ml, which frequently occurred when the ureteropelvic junction was separated. The average postoperative hospital stay was 6 d. There were 6 cases of the single polyp and 1 case of multiple polyps. The polyps of 5 cases were located at the ureteropelvic junction, 1 in the upper ureter and 1 in the middle ureter. The postoperative pathology was consistent with polypoid inflammatory hyperplasia.

The patients had normal diets 24 h after surgery, and no additional complications developed including gastrointestinal motility or mechanical obstruction. The perirenal drainage tube was removed 3 d after surgery, the urinary catheter was removed 4–5 d, and the double J stent was removed 4–6 weeks after surgery. Hematuria occurred in 1 case after surgery. Neither urinary leakage nor urinary tract infection was reported post surgery. Abdominal B ultrasound findings of the hydronephrosis before and 3 months after surgery were compared and used as the standard to evaluate the efficacy of surgery. The affected pyelectasis in this group of patients was measured at 2.0–3.7 cm before surgery whereas 1.2–3.0 cm 3 months after surgery, and no recurrence of polyps was reported. During the follow-up to April 2020, hydronephrosis of all patients was alleviated compared with that before surgery (Table 2).

**Table 2:** Postoperative and follow-up data.

	**Operative time (min)**	**EBL(ml)**	**Hospital stay (days)**	**Polyp site**	**Complications**	**Preoperative hydronephrosis (cm)**	**Postoperative hydronephrosis (cm)**
Patient 1	110	10	9	UPJ	–	2.6	1.2
Patient 2	95	12	9	UPJ	–	3.2	2.0
Patient 3	120	15	10	Upper ureter	Hematuria	3.0	2.2
Patient 4	100	10	8	Middle ureter	–	3.7	3.0
Patient 5	105	15	9	UPJ	–	2.5	1.8
Patient 6	120	10	7	UPJ	–	2.0	1.5
Patient 7	102	10	8	UPJ	–	3.5	2.5

*UPJ, ureteropelvic junction; EBL, estimated blood loss*.

## Discussion

Ureteral polyps represent the most common non-epithelial benign tumors originating from mesoderm, containing fibrous tissue, smooth muscle cells and blood vessels. They have a thick fibrous pedicle and covered with normal transitional epithelium. There is significant smooth muscle thinning, disorder, and fibrosis in the ureteral wall at the attachment of the polyps (4). Fibroepithelial polyps usually originate from the submucosal connective tissue at the proximal ureter and ureteropelvic junction, mostly on the left side (5). The polyps of 5 cases in this group were located at the ureteropelvic junction, 1 in the upper ureter, and 1 in the middle ureter. The postoperative pathology was consistent with polypoid inflammatory hyperplasia. However, the different from the literature was that 4 cases were locally complicated with ureteral muscle hyperplasia and muscle bundle disorder, which was similar to the pathological changes of hydronephrosis caused by ureteropelvic junction stenosis (6).

Compared with hydronephrosis caused by other causes, the onset age of ureteral polyps was later. The age of this group was 7.7–13.9 years old, the average age was 10.4, with relatively mild hydronephrosis, and the damage of renal function was mild. The reason may be that the growth of polyps was slow, the obstruction was mild, and it is not easy to be detected. B-ultrasound, MRU, and renal radionuclide scanning are simple, effective, and reliable techniques for the diagnosis of hydronephrosis nowadays (7). Preoperative MRI revealed ureter or hydronephrosis in 2 cases which were caused by obstruction of soft tissue mass in the ureter. If the ureteral polyp was small in volume and atypical on imaging, it was difficult to confirm the diagnosis as obstructive hydronephrosis caused by ureteral polyps. It is reported that ureteroscopy and retrograde pyelography can improve the positive diagnosis rate of this disease (8). Ureteroscopy and retrograde pyelography in children need to perform general anesthesia and radiation. As both techniques are traumatic examinations, they have the risk of serious urinary tract infection. Based on comprehensive consideration, all cases of this group did not undergo ureteroscopy and retrograde radiography.

The traditional treatment of ureteral polyps is exploration and resection through open surgery, which produces great trauma, more bleeding and long postoperative hospital stay (9). Some literature has reported that holmium laser is used to treat polyps under a ureteroscope. However, due to the narrow field of vision and limited operating space under a ureteroscope, especially in children, it is difficult to distinguish the polyps from the ureteral wall. The surgery is particularly difficult for long segmental polyps or huge polyps, which usually leads to incomplete resection of polyps or ureteral perforation, with high postoperative recurrence. Lam et al. (1) have reported that ureteral polyps are resected through anterograde renal pelvis incision into the ureter using percutaneous ureteroscopy. However, due to the slow growth of polyps and non-malignant nature, the degree of hydronephrosis is usually milder than expected. When underdilated renal pelvis or too narrow UPJ occurs, percutaneous surgery also has some disadvantages of difficulty in ureteroscope insertion, narrow field of vision, limited operating space, incomplete resection of polyps. All 7 children underwent laparoscopic polypectomy, pyeloureterostomy or uretero-ureteral end-to-end anastomosis. The complete polyp in the lumen was visible under laparoscopy, and its size, length, morphology and color were observed. Meanwhile, the polyps and their attached diseased segmental ureter were completely removed to prevent the recurrence of polyps, which can not be completely achieved by endoscopic treatment at present.

As the disease is benign, surgical resection of polyps, pyeloureterostomy or uretero-ureteral end-to-end anastomosis was performed according to the location and type of polyps. The critical point and challenge of laparoscopic treatment of ureteral polyps lie in the anastomosis between the renal pelvis and the normal ureter following the resection of the diseased segmental ureter. The absence of a long segmental ureter refers to the length of ureteral resection ≥ 2 cm. Which is difficult to manage clinically (10). On the one hand, the diseased segmental ureteral is too long; on the other hand, it may be complicated with ureteral stricture, which brings about a challenge for anastomosis. For multiple long segment polyps, it is especially difficult to deal with during surgery. One case in this group developed multiple polyps at an early stage. The first polyp site was located at the ureteropelvic junction during the surgery and the polyp segment ureterectomy and the ureteropelvic anastomosis were performed. Subsequently, the second polyp was found ~2 cm from UPJ. Considering a repeated resection of the diseased segment may be a challenge to the anastomosis, and referring to the method of ureteroscopic treatment for polyps, the second polyp was only treated by the complete resection of polyp to retain the diseased segmental ureter (11). If the diseased segmental ureter is too long, it may lead to excessive tension between the anastomosis and the ureter, resulting in repeated anastomotic obstruction or ureteral necrosis at the anastomotic stoma. According to our experience, if there is a possibility of polyps, we should not immediately dissociate the renal pelvis after dissociating the ureteropelvic junction, when dissociated the ureter downward and located the diseased segment. We should continue to dissociate the distal end of the normal segment of the ureter along the diseased segment, and cut the ureteral diseased segment to find the complete polyp. The difficulty of anastomosis after resection of the polyp segment of the ureter should be evaluated subsequently. If the anastomosis effect is guaranteed, the renal pelvis can be severed, the polyp segment of the ureter can be removed, and the end-to-side anastomosis of the renal pelvis and the normal segment of the ureter can be performed.

Additionally, one case of polyp in this group was located at the upper end of the ureter at 2–3 cm away from the renal pelvis, complicated with stenosis of the proximal ureteropelvic junction. Despite the upper ureteral polyp was complicated with UPJO, no apparent pyelectasis was observed during the surgery, and the ureteropelvic anastomosis was well-free from completed without cutting the renal pelvis first, but retaining the dilated renal pelvis and fully releasing it. Therefore, it is necessary to reserve a dilated renal pelvis flap with appropriate length to coincide with the ureter to reduce the tension of the anastomosis. If the anastomotic tension is still high, the upper renal pelvis flap can be reversed, and the U-shaped flap with the base downward on the anterior wall of the renal pelvis can be used as the extended renal pelvis and anastomose with the distal end of the ureteral incision, which can ensure tension-free anastomotic segment and ureter (12, 13). The author has successfully treated a case of long ureteral stricture applying an inverted renal pelvis flap. Two cases of long segment polyps were included in this group and underwent complete removal of polyps according to the previously described methods, and the anastomosis effect was fully guaranteed. All the children recovered well after surgery, and no complications occurred including anastomotic stricture, urine leakage and ureteral necrosis.

Laparoscopic treatment is a safe, effective and minimally invasive surgical technique for ureteral polyps in childhood. It has benefits of less trauma, clear surgical vision and complete removal of focus, overcoming the shortcomings of the narrow field of vision, difficulty in operations and inability to complete removal of focus using pediatric ureteroscopy. Moreover, compared with open surgery, it also has the advantages of minimally invasive, less trauma, less bleeding, quick recovery, and aesthetic appearance. There are also some limitations in this study, including a small sample cases and non-randomized control grouping. It is expected that further investigations will be conducted with increasing cases accumulated in the future.

## Data Availability Statement

The original contributions presented in the study are included in the article/supplementary material, further inquiries can be directed to the corresponding author/s.

## Ethics Statement

The studies involving human participants were reviewed and approved by research ethics committee of Qilu Hospital of Shandong University. Written informed consent to participate in this study was provided by the participants' legal guardian/next of kin.

## Author Contributions

HG contributed to the design of the study, and drafting the paper. JC and GL contributed to acquisition of data and revision of the paper. XC contributed to analysis and interpretation of data, and revising the paper critically. FS contributed to design, acquisition, analysis, interpretation of data and revising the paper critically. All authors approved the final version of the paper.

## Conflict of Interest

The authors declare that the research was conducted in the absence of any commercial or financial relationships that could be construed as a potential conflict of interest.
